# Acute mesenteric ischaemia

**DOI:** 10.1093/bjs/znad021

**Published:** 2023-02-07

**Authors:** Hanne Fuglseth, Kjetil Søreide, Morten Vetrhus

**Affiliations:** Department of Gastrointestinal Surgery, Stavanger University Hospital, Stavanger, Norway; SAFER Surgery, Surgical Research Group, Stavanger University Hospital, Stavanger, Norway; Department of Gastrointestinal Surgery, Stavanger University Hospital, Stavanger, Norway; SAFER Surgery, Surgical Research Group, Stavanger University Hospital, Stavanger, Norway; Department of Clinical Medicine, University of Bergen, Bergen, Norway; SAFER Surgery, Surgical Research Group, Stavanger University Hospital, Stavanger, Norway; Department of Surgery, Vascular Surgery Unit, Stavanger University Hospital, Stavanger, Norway; Department of Clinical Science, University of Bergen, Bergen, Norway

## Introduction

Acute mesenteric ischaemia (AMI) is a relatively rare but highly lethal condition that requires emergency surgery. It is ranked as one of the most frequent causes of death among surgical diseases on a global level^1^. The estimated incidence is 6–12 events per 100 000 person-years^2,3^, and 1 patient with AMI in about every 2000 hospital admissions. The prevalence of AMI increases with age, with a median age of presentation of around 70 years, and a 10-fold higher risk in octogenarians with co-morbidities compared with the younger population^2,3^. Despite progress in care, the mortality rate is reported to be between 50 and 66 per cent, even in modern series. However, focus on clinical pathways and care bundles can increase awareness, speed up diagnosis, increase revascularization rates, and improve mortality rates compared with historical cohorts.

Three surgical society guidelines^4–6^ exist for AMI, each deviating slightly from the others. The deviations likely depend on both the time of guideline development (from 2016 to 2022) and the various disciplines involved (emergency, gastrointestinal, and vascular surgeons). In addition, a separate set of guidelines^7^ has been provided by a radiology society, involving interventional radiology. Taken together, these recommendations for management highlight the emerging focus on AMI and the multidisciplinary approach to management that has evolved.

## Presentation and clinical signs

**Fig. 1:**
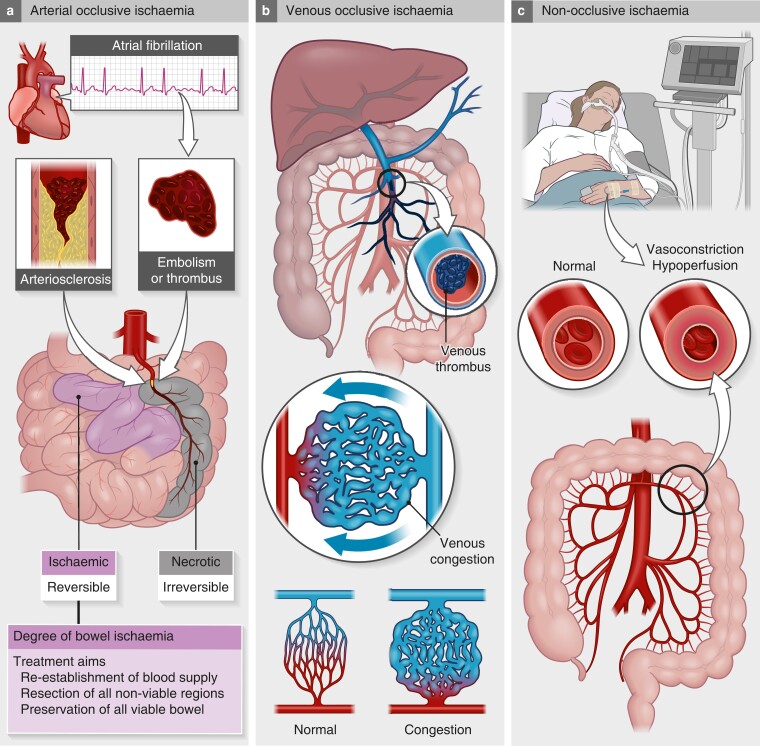
Mechanisms of acute mesenteric ischaemia **a** Arterial occlusion from emboli or thrombus may occur on a background of atherosclerosis. Arterial occlusion is the most frequent cause of acute mesenteric ischaemia, in up to 70 per cent of patients. **b** Mesenteric occlusion is rare, but may occur in up to 20 per cent of patients. **c** Non-occlusive mesenteric ischaemia typically occurs in patients in the ICU or cardiac care unit, owing to hypotension from shock (for example, sepsis) or low cardiac output (for example, heart failure). The frequency of non-occlusive mesenteric ischaemia may be difficult to assess as the underlying condition may prevail over abdominal symptoms, and the correct diagnosis may not be made or suspected.

Sudden onset of abdominal pain with symptoms out of proportion with clinical findings is typical of AMI. However, the presence of clinical symptoms may be more subtle in geriatric patients, and may depend on the underlying mechanism of mesenteric ischaemia (*Fig. 1*).

About 70 per cent of cases of AMI are caused by arterial occlusion^2^. In patients with cardiovascular co-morbidity and atrial fibrillation (known or new-onset fibrillation) and abdominal pain, the suspicion of AMI due to arterial occlusion should be raised (*Fig. 1a*). A high index of clinical suspicion is important for an early diagnosis. As known for other emergency surgical conditions, diagnostic delay resulting from admission under the wrong specialty is also detrimental to outcomes for AMI, as time to intervention is crucial^8,9^.

Mesenteric venous thrombosis is rare and accounts for 10–20 per cent of AMI cases (*Fig. 1b*)^10^. Mesenteric venous thrombosis may be caused by surgery to the upper gastrointestinal tract, and inflammatory conditions, including pancreatitis and thrombophilia. The stagnant venous flow can lead to venous gangrene of the bowel.

Patients with non-occlusive mesenteric ischaemia (NOMI) typically come from the ICU or cardiac care unit^11–13^. NOMI is a result of hypoperfusion (from sepsis, shock, cardiac failure) and use of vasoconstrictors (*Fig. 1c*).

## Diagnostic work-up

**Fig. 2:**
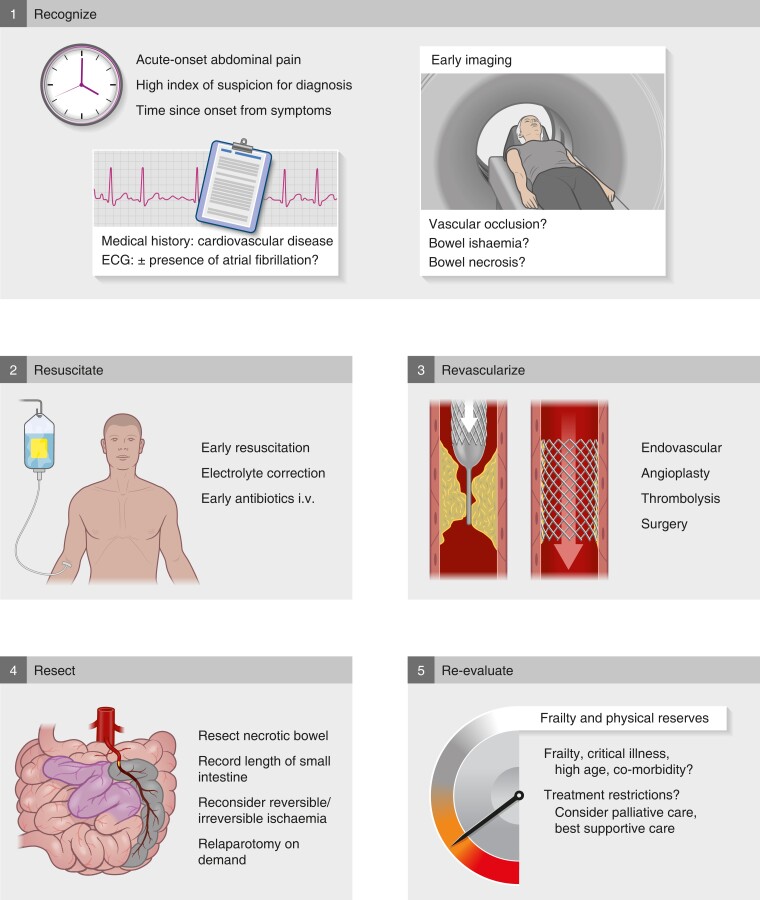
Five key steps in management of acute mesenteric ischaemia i.v., Intravenously.

Appropriate and timely imaging is the most crucial test for early diagnosis of AMI (*Fig. 2*). No currently available blood test is diagnostic of AMI. However, a negative D-dimer test rules out AMI. Plasma citrulline and intestinal fatty acid-binding protein have been proposed as potential biomarkers, but have not made it into clinical use owing to suboptimal accuracy^11,14^. Increased levels of inflammatory markers (white blood cell count, C-reactive protein) may indicate a systemic inflammatory response and systemic effects of bowel ischaemia, as well as translocation of gut bacteria.

Arterial blood gas with lactate is also not diagnostic, and a high lactate level is unfortunately often a late sign of ischaemia. Hence, when clinical suspicion is raised, appropriate imaging should be initiated as an emergency procedure. Cross-sectional imaging with arterial contrast is warranted for rapid assessment. Resuscitation should commence immediately.

CT of the abdomen with intravenous arterial contrast phase is mandatory for diagnosis^4–6,15^. Oral contrast is not needed for AMI assessment. The critical points to investigate include filling of the arteries and any signs of occlusions or arteriosclerosis, as well as assessment of the bowel for ischaemia.

Typical signs of irreversible bowel ischaemia in the presence of AMI are intestinal dilatation and thickness, reduction, or absence of, visceral enhancement, pneumatosis intestinalis, portal venous gas, and free intraperitoneal air^6,16^. Among all radiological signs, bowel wall thinning and portomesenteric venous gas showed highest specificity (98 and 95 per cent) for diagnosis of transmural intestinal infarction^16^.

## Decision-making and strategy

As soon as a diagnosis has been made (or even suspected), early initiation of resuscitation with fluids, correction of electrolytes, and starting intravenous broad-spectrum antibiotics, is warranted (*Fig. 2*).

The patient should be assessed for age, co-morbidity, and physiological reserves. Elderly, frail, and very co-morbid patients may not be surgical candidates, nor candidates for active intervention. In these instances, a purely supportive or palliative approach should be considered after patient consideration and conversation, or discussion with next of kin, as appropriate^17–19^.

For patients who are candidates for intervention, early decision-making and a clear strategy with multidisciplinary discussion (for example, gastrointestinal surgeon, vascular surgeon and interventional radiologist) should be initiated to arrive at the optimal strategy for each patient.

For patients with NOMI (*Fig. 1c*), non-surgical options are needed to correct the problem. The focus is to treat underlying causes, such as stopping or reducing vasopressors, improving fluid balance, and optimizing cardiac output.

## What treatment is appropriate and when?

Patients with occlusive AMI need urgent intervention: first to re-establish blood flow, more specifically to the superior mesenteric artery (SMA), and then to resect non-viable bowel if necessary (*Fig. 2*). The SMA is the most important artery, and the focus is to revascularize it. Owing to the collateral arterial mesenteric network, it is rarely indicated to address both the SMA and the coeliac trunk.

## Open or endovascular approach?

Whether to treat AMI by endovascular or open surgery has not been examined in RCTs, and most of the available literature is based on small, retrospective single-centre studies^5–7,20,21^. However, arguments for an endovascular-first strategy have been put forward for several reasons^21,22^. Endovascular therapy is less invasive than open surgery. Endovascular treatment is performed under local anaesthesia, avoiding general anaesthesia in physiologically compromised or frail patients. In addition, if antegrade recanalization should fail, it does not preclude open surgery. To ensure prompt and complete treatment of arteries and bowel, all endovascular and open surgery for AMI should be performed in a hybrid theatre, if available.

Embolectomy of the SMA is possible by both endovascular and open surgical means, but endovascular therapy has the added possibility of intra-arterial thrombolysis if the patient does not have peritonitis or is in immediate need of laparotomy^23^. Stenting is recommended in thrombotic occlusions, as opposed to plain balloon angioplasty^5^.

If initial endovascular treatment fails, a first step in open operation could be to stent the SMA retrogradely^24^; the alternative is a mesenteric bypass originating from the infrarenal or supracoeliac aorta or the common iliac artery, depending on where and how heavily calcified the arteries are. If the peritoneal cavity is not too contaminated, a synthetic graft is recommended as autologous saphenous vein graft is more prone to kinking.

After revascularization, it must be decided whether there is a need for laparotomy. The guidelines are ambiguous on the merit of laparoscopy^5,6^. One guideline^5^ states that laparoscopy is not a good option as it increases the risk of bowel perforation, and the chances are that it will not be not possible to assess all the distended bowel, whereas another^6^ reports it to be a useful alternative to laparotomy. If the patient has clear peritonitis, a midline laparotomy is indicated. Assessment of bowel viability is based on clinical findings and all devitalized small bowel should be excised, saving as much viable small bowel as possible. If there is uncertainty regarding the viability of the bowel in a physiologically compromised patient, a damage control strategy is recommended, refraining from anastomosing resected bowel, leaving the stapled bowel ends in discontinuity, and planning a second look within 24–48 h. The aim is to preserve as much bowel as possible to avoid short bowel syndrome. Completion angiography should be considered before leaving theatre to assess whether further intervention is needed^22^.

## Treatment of mesenteric venous thrombosis

The aim is to prevent bowel ischaemia, and the first line of treatment is anticoagulation and supportive treatment^10^. Anticoagulation may lead to recanalization in about 80 per cent of patients^25^. Endovascular therapy is an option in patients who do not respond to anticoagulation, including aspiration thrombectomy, thrombolysis, and transjugular intrahepatic portosystemic shunt. Open surgery and bowel resection is needed if the patient continues to deteriorate and develops peritonitis.

## When to keep an open abdomen or plan a second look?

An attempt at closing the abdomen should be the aim^26^, unless specific conditions favour leaving the abdomen open. However, in patients who have undergone a damage control surgical approach, an open abdomen may be a necessity. When leaving an abdomen open, negative-pressure wound therapy with continuous fascial traction is the preferred technique, with the aim of achieving a high delayed fascial closure rate^27^.

## Short- and long-term outcomes

AMI is associated with high mortality rates, reported at around 50–70 per cent^6^. To improve the high mortality rate, early diagnosis and rapid revascularization are essential. A recent single-centre study^28^ from Helsinki University Hospital reported a reduction in 30-day mortality rate to 25 per cent after the implementation of a hospital-specific multidisciplinary pathway and care bundle. The key aspects of the bundle consisted of increased clinical awareness among staff, early use of contrast-enhanced CT, aiming at reduced in-hospital delays, preferred use of hybrid operating theatres, more active revascularization, and increased ICU admission^28^. A large registry study^29^ from North America also pointed to a reduced risk of death and more favourable outcomes in patients selected for endovascular treatment, without establishing any causality between the intervention and outcome.

The long-term mortality rate seems to be only slightly higher than the short-term rate, suggesting that most patients who survive the initial hospital admission for AMI actually have a favourable prognosis^2^. However, there is a lack of data on patient-relevant outcomes other than survival, such as need for parenteral nutrition or presence of a stoma. Extensive bowel resection can result in short bowel syndrome and intestinal failure, and is associated with poor quality of life. Long-term care should focus on minimizing the risk of relapse by treating underlying medical co-morbidity such as diabetes and hypertension. The majority of patients require anticoagulant/antiplatelet therapy^6^.
